# The structural neural substrate of subjective happiness

**DOI:** 10.1038/srep16891

**Published:** 2015-11-20

**Authors:** Wataru Sato, Takanori Kochiyama, Shota Uono, Yasutaka Kubota, Reiko Sawada, Sayaka Yoshimura, Motomi Toichi

**Affiliations:** 1 Department of Neurodevelopmental Psychiatry, Habilitation and Rehabilitation, Graduate School of Medicine, Kyoto University, 53 Shogoin-Kawaharacho, Sakyo, Kyoto 606-8507, Japan; 2 Brain Activity Imaging Center, Advanced Telecommunications Research Institute International, 2-2-2 Hikaridai, Seika-cho, Soraku-gun, Kyoto 619-0288, Japan; 3 Health and Medical Services Center, Shiga University, 1-1-1, Baba, Hikone, Shiga 522-8522, Japan; 4 Faculty of Human Health Science, Graduate School of Medicine, Kyoto University, 53 Shogoin-Kawaharacho, Sakyo-ku, Kyoto 606-8507, Japan; 5 The Organization for Promoting Developmental Disorder Research, 40 Shogoin-Sannocho, Sakyo, Kyoto 606-8392, Japan

## Abstract

Happiness is a subjective experience that is an ultimate goal for humans. Psychological studies have shown that subjective happiness can be measured reliably and consists of emotional and cognitive components. However, the neural substrates of subjective happiness remain unclear. To investigate this issue, we used structural magnetic resonance imaging and questionnaires that assessed subjective happiness, the intensity of positive and negative emotional experiences, and purpose in life. We found a positive relationship between the subjective happiness score and gray matter volume in the right precuneus. Moreover, the same region showed an association with the combined positive and negative emotional intensity and purpose in life scores. Our findings suggest that the precuneus mediates subjective happiness by integrating the emotional and cognitive components of happiness.

Happiness is a subjective experience that has special significance for humans. Several ancient and modern scholars, including Aristotle and Bentham, argued that happiness was the ultimate goal of life^1^. Empirical psychological studies of subjective happiness have shown that the construct can be measured reliably over time^2^, is strongly influenced by genetic factors^3^, and consists of emotional (e.g., experiencing more pleasure and less displeasure) and cognitive (e.g., evaluating one’s life as good) components^1^.

However, the neural substrates of subjective happiness remain unclear. To date, no structural magnetic resonance imaging (MRI) investigation of the construct has been conducted. Identification of the neural substrates underlying subjective happiness may provide a complementary objective measure for this subjective construct and insight into its information-processing mechanism. The psychological evidence showing that subjective happiness can be measured reliably^2^ and is influenced by genetic input^3^ suggests the existence of stable neural substrates.

Previous functional neuroimaging investigations of happiness have consistently found that the induction of happy, as compared to neutral, emotions activated certain brain areas, including the anterior cingulate gyrus^4^,^5^,^6^,^7^,^8^, medial parietal cortex (posterior cingulate gyrus and precuneus)^4^,^5^,^7^,^8^,^9^, and amygdala^4^,^5^,^8^,^9^. These findings are consistent with those of meta-analyses of functional neuroimaging data indicating that these regions are involved in the elicitation of emotional states^10^,^11^,^12^,^13^,^14^. Although the previous studies investigated only the emotional component of subjective happiness, we hypothesized that the neural correlates of subjective happiness would include these brain regions. Moreover, we hypothesized that the emotional- and cognitive-component model of subjective happiness would be valid at the neural level.

We used structural MRI and measures of subjective happiness^2^, and possible emotional (positive and negative emotional intensity^15^), and cognitive (the evaluation of purpose in one’s life^16^) components of happiness to test these hypotheses. We also assessed participants’ trait and state anxiety level^17^ to confirm the validity of subjective happiness as a stable construct with a prediction that the subjective happiness score would be negatively correlated with trait^18^, but not state, anxiety.

## Results

### Psychological rating

The means ± standard deviations (*SD*s) of the psychological rating scores are shown in Table 1. The subjective happiness score (mean ± *SD*, 4.6 ± 0.8) was in good agreement with that of a previous standardization study^19^ (mean ± *SD*, 4.7 ± 1.1).

The psychological model positing that subjective happiness consists of emotional and cognitive components was tested using a multiple regression analysis. The regression equation contained the subjective happiness score as the dependent variable and the intensity of positive and negative emotions and purpose in life as the effect-of-interest independent variables. Sex, age, and full-scale intelligence quotient (IQ) were also included as covariates (effects of no interest). The results revealed a significant association between the subjective happiness score and the combined intensity of positive and negative emotions and purpose in life scores (*F*(6, 44) = 4.37, *p* < 0.005). The parameter estimate profile revealed that subjective happiness was positively associated with positive emotional intensity (β = 0.12) and purpose in life (β = 0.43) and negatively associated with negative emotional intensity (β = −0.28).

We assessed the relationship between the subjective happiness score and the trait and state anxiety scores to confirm the convergent and discriminant validity of the subjective happiness score. The subjective happiness score was significantly negatively correlated with the trait (*r* = −0.52, *p* < 0.001), but not state (*r* = 0.03, *p *>* *0.1), anxiety score.

### Gray matter volume associated with subjective happiness

**Figure 1:**
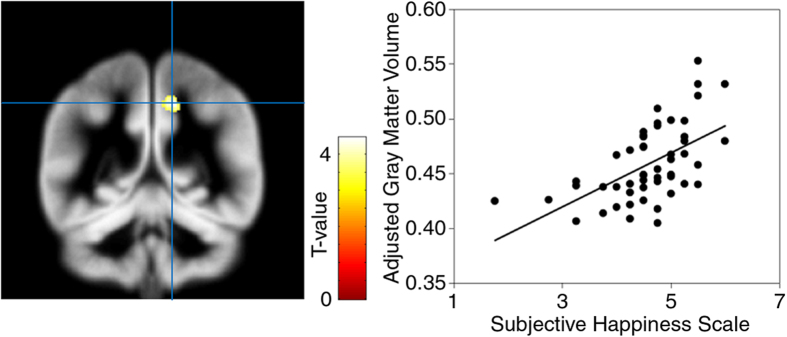
Brain region significantly associated with the subjective happiness score. (Left) A statistical parametric map (*p* < 0.001, peak-level uncorrected for display purposes). The area is overlaid on the spatially normalized gray matter tissue probability map. The blue cross indicates the location of the peak voxel. The red-white color scale indicates the *T*-value. (Right) A scatter plot of the adjusted gray matter volume as a function of the subjective happiness score at the peak voxel.

To identify the brain regions associated with subjective happiness, the structural MRI data were analyzed using a multiple regression analysis with the subjective happiness score as the independent variable and sex, age, and full-scale IQ as covariates. The results revealed a significant positive relationship between the subjective happiness score and gray matter volume in the right precuneus (x14, y-48, z46; *T*(46) = 4.44, *p* < 0.05, peak-level family-wise error (FWE) corrected within regions of interest (ROIs); cluster size = 222.8 mm^3^; Fig. 1). The subjective happiness score was not significantly associated with any other brain region (*p* < 0.05, peak-level FWE-corrected across the whole brain). An exploratory analysis using a more liberal threshold (*p* < 0.001, peak-level uncorrected) also revealed the same results. The exploratory analysis investigating a negative relationship between subjective happiness and gray matter volume with this liberal threshold revealed small (*p* > 0.1, cluster-level uncorrected) clusters in the regions around the cerebellum (x-53, y-30, z-29), superior frontal gyrus (x-15, y27, z64), and insular cortex (x-30, y17, z24) in the left hemisphere.

### Gray matter volume associated with the combined positive and negative emotion intensity and purpose in life scores

**Figure 2:**
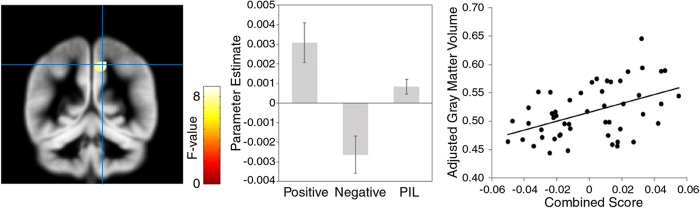
Brain region significantly associated with the combined positive and negative emotional intensity and purpose in life scores. (Left) A statistical parametric map (*p* < 0.001, peak-level uncorrected for display purposes). The area is rendered on the spatially normalized gray matter tissue probability map. The blue cross indicates the location of the peak voxel. The red-white color scale indicates the *F*-value. (Center) Parameter estimates (with standard errors) for the regressors relating to positive and negative emotion intensity and purpose in life (PIL) scores at the peak voxel. Positive and negative values indicate positive and negative associations with gray matter volume, respectively, in arbitrary units. (Right) A scatter plot of adjusted gray matter volume as a function of the combined scores at the peak voxel. The combination scores were calculated by weighted-summing three (mean centered) regressors of interest, each multiplied by the associated parameter estimates at the peak coordinates in the right precuneus.

To identify the brain regions associated with the combined emotional and cognitive components of happiness, we performed a multiple regression analysis on the MRI data with the positive and negative emotional intensity and purpose of life scores as independent variables and sex, age, and full-scale IQ as covariates. A significant association was found between the combined positive and negative emotional intensity and purpose in life scores and gray matter volume in the right precuneus (x12, y-51, z48; *F*(3, 44) = 8.60, *p* < 0.05, peak-level FWE-corrected within ROIs; cluster size = 178.9 mm^3^; Fig. 2), which overlapped with the cluster showing an association with the subjective happiness score. The parameter estimate profile revealed that gray matter volume was positively correlated with positive emotional intensity and purpose in life and negatively correlated with negative emotional intensity (β = 0.43, 0.31, and –0.40, respectively; *p* < 0.05, peak-level uncorrected). The overlap in the right precuneus between the subjective happiness and the combined scores was confirmed using a conjunction analysis (x12, y-48, z46, *T*^2^(96) = 3.96, *p* < 0.05, peak-level FWE-corrected within ROIs; cluster size = 168.8 mm^3^).

## Discussion

Our analysis of psychological ratings revealed that the mean subjective happiness score was similar to that reported in a previous study^19^. The correlation analysis revealed that subjective happiness was negatively associated with negative emotional traits, as previously reported^18^, but not with the negative emotional states. The regression analysis revealed that the subjective happiness score could be explained by the combined intensity of positive and negative emotions and purpose in life scores. Our results support previous theoretical and empirical data^1^ and indicate that subjective happiness is a stable construct consisting of emotional and cognitive components.

More important, our analysis of structural MRI data revealed that the subjective happiness score was associated with increased gray matter volume in the precuneus. This finding is largely in line with those of previous functional neuroimaging studies showing that the induction of happy emotional states was associated with activation of the medial parietal cortex^4^,^5^,^7^,^8^,^9^. However, these previous studies investigated a transiently induced happy mood state, whereas subjective happiness, which is a more complex and stable subjective experience, has not been evaluated. Involvement of the precuneus in subjective happiness is consistent with empirical and theoretical findings regarding this region. Previous functional neuroimaging studies have shown that the precuneus region has the highest level of cortical glucose metabolism in the brain, highlighting the importance of this region for subjective consciousness in humans^20^. Several neuroimaging studies revealed that the precuneus is involved in changes in subjective experience (e.g., the gradual transition of consciousness level following the induction of anesthesia^21^,^22^), suggesting that this region may be involved in the production and/or alteration of subjective experiences^20^,^23^. Although this psychological function fits well with the concept of subjective happiness, no previous study investigated precuneus involvement in subjective happiness. To our knowledge, our study is the first to show that the precuneus is associated with subjective happiness.

Furthermore, our results revealed an association between the precuneus and the combined positive and negative emotional intensity and purpose in life scores. These results indicate that the widely accepted psychological model postulating emotional and cognitive components of subjective happiness^24^ may be applicable at the level of neural structure. A previous neuroanatomical study in monkeys has found that the precuneus receives projections from widespread cortical and subcortical regions^25^. Several neuroimaging studies in humans also revealed that the precuneus is involved in the default mode network and communicates with widespread brain regions^22^,^26^. Moreover, the precuneus is involved in self-referential processing, which integrates the information of one’s current internal experience, past memory, and future plans^20^,^27^,^28^. Thus, the region is thought to be involved in the integration of internal and external information^22^. Together with these findings, our results suggest that the precuneus may play an important role in integrating different types of information and converting it into subjective happiness.

Our finding that subjective happiness can be predicted using structural measurements has practical implications. Interest in subjective happiness has increased as its importance as the “the ultimate currency” and association with life success and good physical health has been highlighted by recent psychological studies^29^. In terms of public policy, subjective happiness is thought to be a better indicator of happiness than economic success^30^. However, the subjective measures of happiness have inherent limitations, such as the imprecise nature of comparing data across different cultures and the difficulties associated with the applications of these measures to specific populations, including the intellectually disabled^31^. Our results show that structural neuroimaging may serve as a complementary objective measure of subjective happiness.

Although our results provide evidence for the existence of a neural substrate underlying subjective happiness, they do not show that the construct is unchangeable. On the contrary, previous structural neuroimaging studies have shown that training in psychological activities, such as meditation, changed the structure of the precuneus gray matter^32^. These findings are consistent with those of previous studies showing that meditation training increased subjective happiness^33^. Together with these findings, our results suggest that psychological training that effectively increases gray matter volume in the precuneus may enhance subjective happiness.

Our study has several limitations, some of which may account for the discrepancies between our results and those of previous studies. First, although the precuneus was the only brain region significantly associated with subjective happiness in our study, several previous functional imaging studies found that other brain regions, such as the anterior cingulate gyrus and amygdala, were active during the induction of happy emotions^4^,^5^,^6^,^7^,^8^,^9^. This disparity may be explained by the methodological differences among the studies when measuring hemodynamic responses versus gray matter volumes. Alternatively, it may be that our small sample size lacked the power to detect an association of subjective happiness with other brain regions. Consistent with this notion, we found non-significant negative associations between subjective happiness and various brain regions, such as the insular cortex, previously reported to be involved in negative emotional states^10^. Future studies with a larger sample size may reveal the involvement of other brain regions and neural networks in the experience of subjective happiness.

Second, although we found that the cognitive and emotional components of happiness were associated with precuneus volume, recent structural MRI studies using a different methodology to investigate the structural neural substrates of the cognitive component of happiness reported different findings^34^,^35^. Specifically, these studies found that eudaimonic well-being^36^ was positively associated with right insular cortex volume^35^ and that life satisfaction^37^ was positively associated with right parahippocampal gyrus volume and negatively associated with left ventromedial prefrontal cortex and left precuneus volumes^34^. We speculate that the cognitive component of happiness may contain multiple subcomponents and our use of a single purpose-of-life measure may not tap into all of these subcomponents. Further studies are needed to clarify the relationships between cognitive subcomponents of happiness and their associations with structural neural substrates.

Finally, the present study assessed the psychological ratings of the participants after the MRI scans and this procedure may not have been ideal for the measurement of subjective happiness as a stable construct. It is possible that the MRI acquisition procedure altered the transient mood of some participants which, in turn, had a confounding effect on the subjective happiness ratings. Future research that measures subjective happiness under calm conditions independently from MRI scanning would be preferable for rigorously examining the structural neural substrates of subjective happiness.

In conclusion, our investigation using structural MRI and questionnaires revealed a positive relationship between the subjective happiness score and gray matter volume in the right precuneus. Furthermore, we found a correlation between the combined purpose in life and positive and negative emotional intensity scores and gray matter volume in the same region. Our findings suggest that the precuneus mediates subjective happiness by integrating the emotional and cognitive components of happiness.

## Methods

### Participants

The study included 51 volunteers (26 females; mean ± *SD* age, 22.5 ± 4.5 years). The participants were administered the Mini-International Neuropsychiatric Interview^38^, a short structured diagnostic interview, by a psychiatrist or psychologist. The interview revealed no neuropsychiatric conditions among participants. All participants were right-handed, as assessed by the Edinburgh Handedness Inventory^39^. After a detailed explanation of the experimental procedure, all participants provided informed consent. Our study was approved by the ethics committee of the Primate Research Institute, Kyoto University. The study was conducted in accordance with the Declaration of Helsinki.

### Psychological questionnaires

The Japanese version of the Subjective Happiness Scale^2^,^19^, a 4-item measure of global subjective happiness, was used to measure the subjective happiness of the participants. The reliability and validity of the questionnaire has been verified in Japanese participants^19^. We used the Japanese version of the Emotional Intensity Scale^16^,^40^ to assess the intensity of positive and negative emotions. The reliability and validity of this scale has been verified in Japanese participants^40^. The cognitive component of happiness was measured using the Japanese version of the Purpose in Life Test (A scale)^20^,^41^, the reliability and validity of which has been verified previously in Japanese participants^41^. Although previous studies^42^ have considered life satisfaction to be a cognitive component of happiness and measured it using the Satisfaction with Life Scale^37^, we did not use this scale because our preliminary investigation revealed a high correlation between the scores of this scale and those of the Subjective Happiness Scale (*r* > 0.7), which was consistent with previous samples^2^, and because a previous study found that ‘meaning in life’ is a substantial component of ‘life satisfaction’ among Japanese participants^43^. The Japanese version of the State-Trait Anxiety Inventory^17^,^44^ was used to measure state and trait anxiety. The reliability and validity of the inventory has been verified in Japanese participants^44^. The present study was part of a larger project investigating personalities and mental health.

### Procedure

Each participant underwent MRI and then completed a set of questionnaires.

### MRI acquisition

Image scanning was performed using a 3-Tesla scanning system (MAGNETOM Trio, A Tim System; Siemens) using a 12-channel head coil. A forehead pad was used to stabilize the head position. A T1-weighted high-resolution anatomical image was obtained using magnetization-prepared rapid-acquisition gradient-echo sequence (repetition time = 2250 ms; echo time = 3.06 ms; inversion time = 1000 ms; flip angle = 9° field of view = 256 × 256 mm; voxel size = 1 × 1 × 1 mm).

### Psychological data analysis

The data were analyzed using SPSS 16.0 J (SPSS Japan). We performed a multiple regression analysis using the subjective happiness score as the dependent variable and the intensity of positive and negative emotions and purpose in life scores as the effect-of-interest independent variables. Sex, age, and full-scale IQ (measured using the Wechsler Adult Intelligence Scale-Third Edition) were also included as covariates. The significance of association was evaluated using *F*-statistics. We also analyzed correlations between the subjective happiness score and state/trait anxiety scores. The significance of correlation coefficients was evaluated using *t*-statistics (two-tailed). The results of all tests were considered statistically significant at *p* < 0.05.

### Image analysis

Image analyses were performed using the SPM8 statistical parametric mapping package (http://www.fil.ion.ucl.ac.uk/spm) and the VBM8 toolbox (http://dbm.neuro.uni-jena.de) implemented in MATLAB R2012b (Mathworks). First, image preprocessing was performed using the VBM8 toolbox using the default settings. All structural T1 images were segmented into gray matter, white matter, and cerebrospinal fluid using an adaptive maximum a posteriori approach^45^. Intensity inhomogeneity in the MRI was modeled as slowly varying spatial functions, and thus corrected in the estimation. The segmented images were then used for a partial volume estimation using a simple model with mixed tissue types to improve segmentation^46^. Furthermore, a spatially adaptive non-local means denoising filter was applied to deal with spatially varying noise levels^47^. A Markov Random Field cleanup was used to improve the image quality. The gray and white matter images in native space were subsequently normalized into standard stereotactic space defined by the Montreal Neurological Institute using the diffeomorphic anatomical registration using the exponentiated Lie algebra algorithm approach^48^. We used the predefined templates provided with the VBM8 toolbox, derived from 550 healthy brains from the IXI-database (http://www.brain-development.org). The resulting normalized gray matter images were modulated using Jacobian determinants with non-linear warping only (i.e., m0 image in VBM8 outputs) to exclude the effect of total intracranial volume. Finally, the normalized modulated gray matter images were resampled to a resolution of 1.5 × 1.5 × 1.5 mm and smoothed using an isotropic Gaussian kernel 12-mm full width at half-maximum to compensate for anatomical variability among participants.

We performed a multiple regression analysis using the subjective happiness score as the independent variable and sex, age, and full-scale IQ as covariates. The positive relationship between subjective happiness and gray matter volume was tested using *t*-statistics. We also performed a multiple regression analysis using the intensity of positive and negative emotions and purpose in life scores as the independent variables and sex, age, and full-scale IQ as covariates; the effect of the combined score was assessed using *F*-statistics. Finally, a conjunction analysis using the subjective happiness and combined scores derived from the regression analyses was performed to statistically assess the commonalities among these effects, and the contrast was evaluated using minimum t-statistics compared to the conjunction null^49^. For the ROIs described in the Introduction, a small volume correction^50^ was performed for the union of anatomical masks. The anatomical masks of the 12-mm radius spheres were centered on the activation foci in the left anterior cingulate gyrus (x-4, y34, z-8), left posterior cingulate gyrus (x-4, y-50, z22), right precuneus (x16, y-44, z56), and left amygdala (x-30, y-30, z-20). We selected these ROIs because a previous study, which compared happy versus neutral emotional induction, analyzed the whole brain, and reported the coordinates of activation foci, found significant activation during happy emotion induction in these regions^5^. Voxels were deemed to be statistically significant if they reached the height threshold of *p* < 0.05, peak-level FWE-corrected for multiple comparisons within the ROIs. Other areas were *p* < 0.05, peak-level FWE-corrected for the entire brain volume. For exploratory purposes, we performed the analysis using a liberal height threshold of *p* < 0.001 (peak-level uncorrected) and tests of a negative relationship for the subjective happiness score. The brain structures were labeled anatomically using Talairach Client (http://www.talairach.org/)^51^ and Automated Anatomical Labeling atlas^52^ included in the MRIcron software (http://www.mccauslandcenter.sc.edu/mricro/mricron/).

The relationships between gray matter volumes and psychological measures (subjective happiness score or combined positive and negative emotion intensity and purpose in life scores) were illustrated by plotting the gray matter values extracted at the peak voxels against the psychological measures after adjusting for the effects of no interest by regressing out sex-, age-, and IQ-related variance. To create the combination score, each regressor of interest was multiplied by its parameter estimates and these products were summed across the three regressors of interest.

## Additional Information

**How to cite this article**: Sato, W. *et al.* The structural neural substrate of subjective happiness. *Sci. Rep.* **5**, 16891; doi: 10.1038/srep16891 (2015).

## Figures and Tables

**Table 1:** Mean (with standard deviation) data of psychological ratings.

Subjective Happiness	Positive Emotion	Negative Emotion	Purpose in Life	State Anxiety	Trait Anxiety
4.6 (0.8)	49.9 (6.7)	48.9 (7.4)	90.9 (18.0)	39.1 (5.8)	46.3 (8.6)
